# Evaluation of a dressing film for ultrasound-guided vascular puncture to achieve high-quality imaging and infection prevention

**DOI:** 10.1007/s40477-025-01067-y

**Published:** 2025-08-31

**Authors:** Mari Abe, Toshiaki Takahashi, Miyako Muta, Atsuo Kawamoto, Ryoko Murayama, Gojiro Nakagami

**Affiliations:** 1 Research Institute for Next-Generation Nursing Education, Tokyo, Japan; 2 Department of Nursing, The University of Tokyo Hospital, Tokyo, Japan; 3 Department of Gerontological Nursing/Wound Care Management, Graduate School of Medicine, The University of Tokyo, Tokyo, Japan; 4 Department of Diagnostic Imaging, Tokyo Medical University Hospital, Tokyo, Japan; 5 Faculty of Nursing, School of Health Sciences, Fujita Health University, Aichi, Japan; 6 Global Nursing Research Center, Graduate School of Medicine, The University of Tokyo, Tokyo, Japan

**Keywords:** Forearm, Infection control, Punctures, Radial artery, Peripheral vascular access

## Abstract

**Purpose:**

This study evaluated the quality of ultrasound images obtained during peripheral vascular catheter insertion using a transparent film designed to maintain puncture site sterility during ultrasound-guided puncture.

**Methods:**

Images were collected from 10 healthy adult participants with and without film, focusing on the radial artery, forearm cephalic vein, and median cubital vein. In total, 300 ultrasound still images were assessed using a 10-point Likert scale.

**Results:**

Image quality was significantly lower at all sites with the film (mean total image quality: radial artery, 5.2 vs. 6.0: *p* = 0.019; forearm cephalic vein, 6.1 vs. 7.6: *p* < 0.001; median cubital vein, 6.0 vs. 7.4: *p* < 0.001). However, the clinical nurse’s evaluation of puncture feasibility showed no significant difference for the radial artery (80.0% vs 96.7%) and forearm cephalic vein (100.0% vs 100.0%).

**Conclusion:**

Compromised image quality using the film does not negatively affect the puncturability of the radial artery and forearm veins. This finding underscores the potential for maintaining sterile conditions during procedures without compromising the ability to successfully perform puncture, thereby improving patient outcomes and procedural efficiency.

## Introduction

The ultrasound technique for peripheral vessel catheter placement is gaining widespread use [1], as it can easily visualize the vessel diameter, depth, and vessel health status, including the presence of thrombi and subcutaneous edema surrounding the vein [2–4]. This information is important for safe catheterization from the viewpoint of increasing the success rate and preventing catheter failure because of complications after catheter placement [1, 5].

To obtain high-quality ultrasound images, it is crucial to adequately fill the gap between the probe and the skin surface with ultrasound gel. To visualize the vessel and needle tip during puncture, the puncture point—specifically, the needle tip—must be positioned near to the ultrasound probe. The puncture site should be kept clean to prevent infection, and a sterile gel or probe cover is required for sterility. This approach incurs both cost and effort. As an alternative to the use of sterile gel or probe cover, a disinfectant solution can sometimes be used in place of ultrasound gel. However, the use of disinfectant limits the ability to properly angle the probe, and successful puncture requires a high level of technical skill. In particular, for beginners, focusing on the ultrasound display during guided puncture procedures while also ensuring that the needle tip does not contact the probe and becomes contaminated can be challenging.

To address these clinical challenges, a dressing film designed for ultrasound-guided puncture was developed. This film allows for puncture while maintaining puncture site sterility [6]. A previous study was a preliminary investigation, reporting the evaluation of a single case involving the basilic vein of the upper arm. Given the need to assess sites frequently accessed for treatment or diagnostic purposes in clinical practice, this study aimed to evaluate the image quality obtained through the film used in commonly catheterized sites, such as the radial artery, forearm cephalic vein, and median cubital vein. Furthermore, puncture feasibility was assessed by a nurse who performed ultrasound for peripheral vascular catheterization in clinical practice.

## Materials and methods

### Study design and setting

This cross-sectional observational study examined whether a significant difference exists in images obtained with and without the film. This study was conducted at the research laboratory of The University of Tokyo in June 2024.

### Data collection procedure

Participants were recruited using a snowball sampling method. The inclusion criteria were adults aged ≥ 20 years, and the exclusion criteria were individuals with skin diseases. After collecting age and sex information, the participants were assigned a research ID. Data collection sites included the bilateral forearm cephalic veins, bilateral median cubital veins, and right radial artery, totaling five locations. The order of data collection was randomly determined using a random number table. The reason for not obtaining images from the left radial artery was that it is rarely used during cardiac catheterization procedures or catheter treatments. For each site, three short-axis ultrasound still images were obtained for cases with and without films. In total, 30 ultrasound images were obtained from each participant. The ultrasound system was a smartphone-type device with a linear probe of 5–10 MHz (iViz air, FUW-1; Fujifilm, Tokyo, Japan), which was generally used in clinical settings for peripheral vascular access.

### Film for ultrasound-guided puncture

**Fig. 1:**
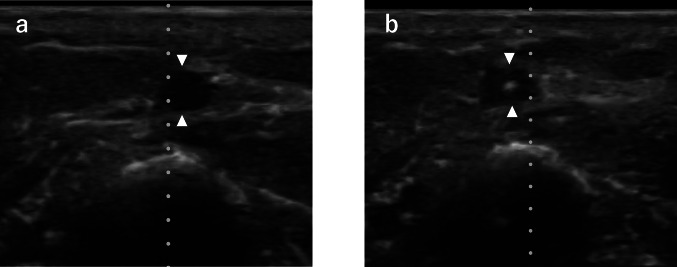
Ultrasound images acquired through the film in a clinical setting. **a** Transverse image of the target vein for puncture: arrowheads indicate the vessel. **b** Transverse image of the vein during puncture: arrowheads indicate the vessel, and the bright spot within the vessel is the needle tip

The film is commercially available (CATHEREEPLUS_TM_ECHO CPSE0810; Nichiban Corporation, Tokyo, Japan) and has already been used clinically in patients undergoing peripheral intravenous catheter placement (Fig. 1).

**Fig. 2:**
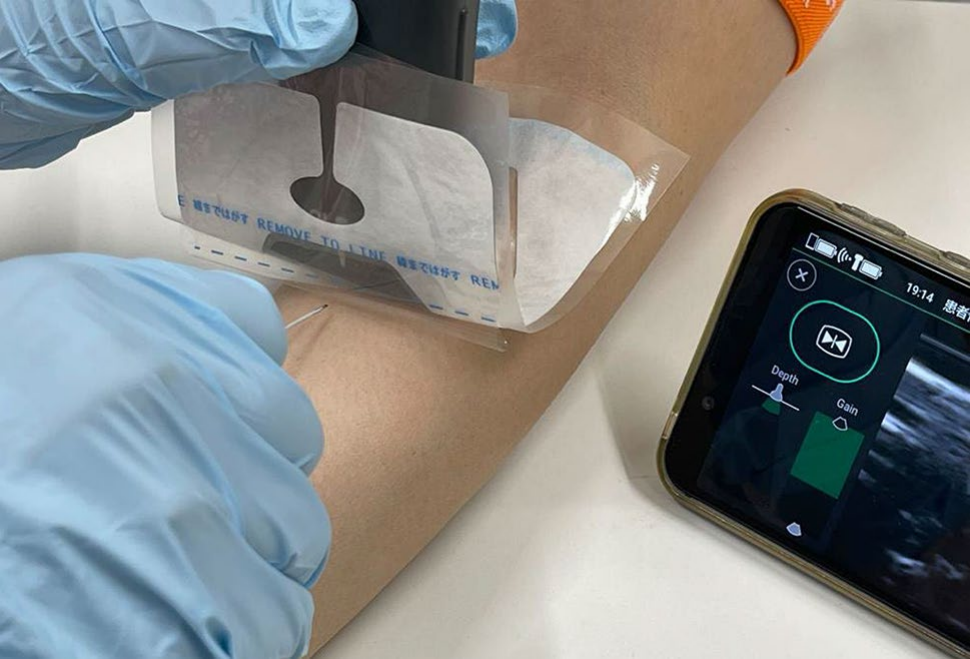
The scene of performing a puncture using film for ultrasound-guided puncture. A sterile film is placed between the probe and the needle, preventing direct contact between them. Since the film is sterile, even if the needle accidentally touches the film, it will not compromise sterility

It consists of three-layer films. One layer of the film at the deepest layer (closest to the skin surface) is partially peeled away (about half) and adhered to the skin, with the ultrasound probe placed on top after applying ultrasound gel (Fig. 2). In this way, images are obtained through two layers of film. The top layer (side facing the probe) was designed to be removable along with the ultrasound gel. The remaining film (middle layer) is used to secure the catheter [6].

### Ultrasound operator and image acquisition sites

All ultrasound images were obtained by a single operator with 3 years of clinical experience and > 5 years of research experience related to the use of ultrasound for peripheral venous catheter placement. The operator positioned the probe over the radial artery at the site where arterial pulsation was palpated, and the location considered most suitable for catheter insertion was selected. Similarly, for the median cubital and forearm cephalic veins, imaging was performed at sites deemed optimal for peripheral venous catheter placement. The cephalic vein of the forearm was examined between the wrist and elbow joints; the researcher captured images at locations along the proximal half that were considered appropriate for catheter placement.

### Ultrasound image evaluation

1. Ultrasonographer.
  An ultrasonographer conducted a blinded assessment of the ultrasound image quality, evaluating the following criteria: a) image resolution (RES), b) detail (DET), and c) total image quality (TIQ), using a 10-point Likert scale. RES was defined as the sharpness and crispness of the image and a lack of haziness/blurriness. DET was defined as the clarity of organ outlines and ease with which the boundaries of structures are seen and how well they are defined. TIQ was defined as an overall assessment encompassing the contrast between solid and fluid-filled structures and absence of noise. The evaluation method was based on a previous study that assessed image quality using two different ultrasound devices [7, 8]. The sonographer evaluated the image quality of the blood vessels and surrounding tissue within approximately 1.0 cm. The image resolution for evaluation was 1598 by 1080 pixels.
2. Clinical nurse.
  A nurse who had performed > 300 catheter placements using ultrasound in a clinical setting evaluated the images obtained to determine whether puncture for peripheral vessel canulation was possible. The images used for evaluation were the same as those presented to the ultrasound technicians, with a resolution of 1598 by 1080 pixels.

### Data analysis

The RES, DET, and TIQ scores were examined and compared with and without film. The paired t-test was used to compare image quality. Fisher’s exact test was used to compare puncturability assessed by a clinical nurse between the two groups. The significance level was set at 5%. All statistical data were analyzed using JMP version 17.0 (SAS Institute Inc., USA).

### Ethical considerations

Before data collection, participants received an explanation from a researcher and provided written informed consent. The study protocol was approved by the Research Ethics Committee of the Faculty of Medicine, The University of Tokyo (No. 2023248NI).

## Results

The participants consisted of 10 individuals. The mean age of the participants was 33.4 (standard deviation, 7.3) years, with seven females (70.0%). In total, 300 ultrasound static images were obtained, including 120 images of the bilateral forearm cephalic veins, 120 of the bilateral median cubital veins, and 60 of the right radial arteries.

**Fig. 3:**
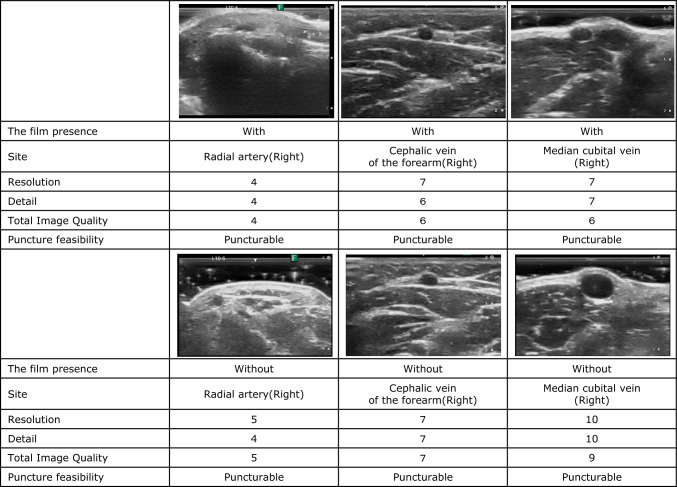
Image and evaluation examples with and without the film. The images above and below are from the same individual and the same anatomical site

Image quality was significantly lower at all three locations with film than that without film. The mean TIQ score for each site was calculated as follows: radial artery (with vs. without film, 5.2 vs. 6.0, *p* = 0.002), forearm cephalic vein (with vs. without film, 6.1 vs. 7.6, *p* < 0.001), and median cubital vein (with vs. without film, 6.0 vs. 7.4, *p* < 0.001) (Table 1; Fig. 3).

**Table 1:** Comparison of the ultrasound image quality with and without the film

	Image through film		Image without film		*p*-value
	Mean	95%CI	Mean	95%CI	
Right radial artery (*n* = 60)					
Resolution	5.5	5.2–5.9	6.1	5.8–6.4	0.019
Detail	4.9	4.5–5.3	5.9	5.5–6.3	0.001
Total image quality	5.2	4.9–5.5	6.0	5.6–6.3	0.002
Forearm cephalic vein (*n* = 120)					
Resolution	6.4	6.2–6.7	7.5	7.2–7.7	< 0.001
Detail	6.2	5.9–6.5	7.5	7.2–7.8	< 0.001
Total image quality	6.1	5.8–6.4	7.6	7.3–7.9	< 0.001
Median cubital vein (*n* = 120)					
Resolution	6.3	6.1–6.5	7.5	7.3–7.8	< 0.001
Detail	6.1	5.8–6.4	7.6	7.2–7.9	< 0.001
Total image quality	6.0	5.7–6.4	7.4	7.1–7.6	< 0.001

*CI* confidence interval

**Fig. 4:**
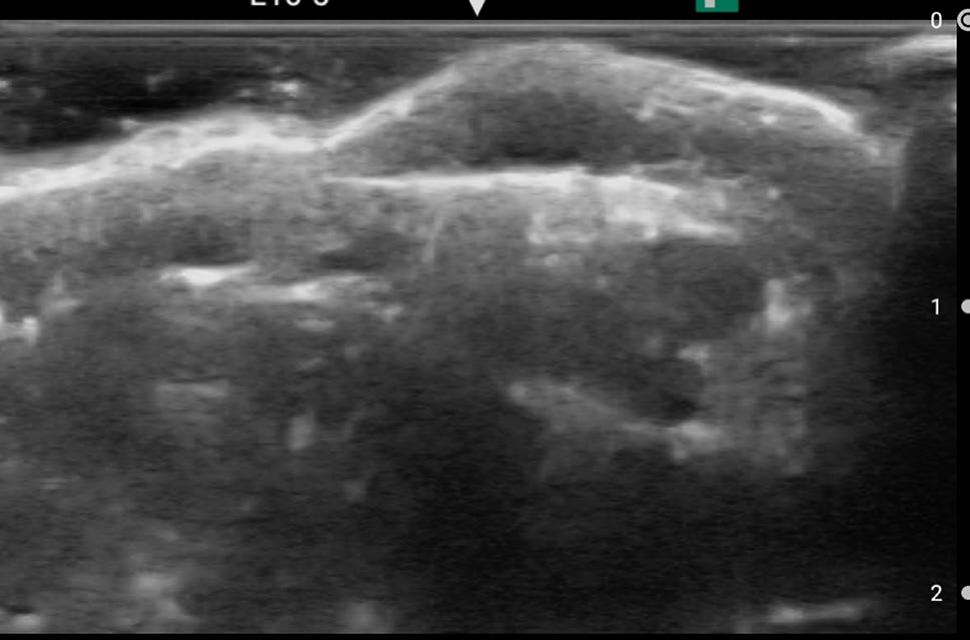
An ultrasound image which was evaluated as non-puncturable by a clinical nurse. The image was taken at right median cubital vein with the film

Puncturability assessed by nurses showed the following results: radial artery (with vs without film), 80.0% vs 96.7% (24/30 vs 29/30, *p* = 0.103); forearm cephalic vein, 100.0% vs 100.0% (60/60 vs 60/60, *p* = 1.000); and median cubital vein, 86.7% vs 100.0% (52/60 vs 60/60, *p* = 0.006). Figure 4 shows an ultrasound image of the cubital vein, which was evaluated as non-puncturable by a clinical nurse.

## Discussion

This study evaluated the quality of ultrasound images obtained using a specialized film for ultrasound-guided puncture. The image quality scores were significantly higher without film than with film at all three sites (radial artery, forearm cephalic vein, and median cubital vein). Conversely, in the clinical evaluation of puncturability by nurses, no significant differences were observed between cases with and without film for the radial artery and forearm cephalic vein.

Overall, the lower image quality observed with film is likely due to the scattering that occurs when ultrasound waves pass through different media [6, 9]. The film may have caused the scattering of the ultrasound beam, potentially affecting image resolution and quality. Scattering is more likely to occur at the boundary between the film and surrounding tissues because of differences in the acoustic impedance. In addition, scattering may have been caused not only by the mismatch in acoustic impedance between the film and the skin, but also by the incomplete adhesion of the film to the skin, potentially leaving air between the two surfaces. Furthermore, the film is composed of three layers, each with different acoustic impedance, which may also have contributed to the scattering.

This could explain the lower image quality with film than without film. Attenuation, in which the intensity of ultrasound waves decreases as they pass through tissues, also occurs. The focus of this evaluation was the assessment of superficial blood vessels. Given the short distance through the tissues through which the ultrasound waves traveled, the effect of attenuation was considered minimal.

In the assessment of whether clinical nurses could perform vessel puncture, the superficial veins in the antecubital fossa were significantly more often deemed unsuitable for puncture in the group with film application. Nurses’ criteria for determining whether vessel puncture is feasible are based on the clarity of the depiction of the vessel walls on ultrasound imaging. The antecubital fossa typically exhibits multiple superficial veins, including the cephalic, basilic, and median cubital veins. Owing to the proximity and prominence of these veins to the skin surface, the film may not adhere well to the skin in that area, potentially making it difficult to achieve a clear depiction of the vessel walls.

Although the use of peripheral venous catheters in the cubital fossa is generally deemed inappropriate in the clinical guidelines [10], reports on such practices remain prevalent [11]. Accordingly, the median cubital vein was selected as the puncture site in this study. The results revealed that film use significantly increased the risk of the nonpuncturability of the median cubital vein. However, the finding that the film did not influence the feasibility of puncturing the cephalic vein in the forearm—a site recognized as optimal for catheter placement—provides a positive perspective for clinicians. The reason is that the cubital vein is used as a site for peripheral venous catheter placement because vascular access is difficult. Ultrasonography has already been shown to be effective in addressing challenges related to difficult venous access [12], and the increased adoption of ultrasound-guided peripheral venous catheter placement may increase catheterization at the forearm, which is regarded as a more appropriate site. Therefore, the feasibility of forearm cephalic vein puncture with a film suggests that its application could promote ultrasound-guided puncture in clinical practice.

However, this study has some limitations. First, the number of participants was small, and the study population consisted mainly of young and healthy individuals, as the only exclusion criterion was the presence of skin diseases. This does not adequately represent the general patient population, particularly hospitalized patients, who are often older and have comorbidities that may affect both image quality and vascular puncturability. Second, the nurse evaluated saved still images; however, healthcare providers puncture the blood vessel with real-time ultrasound images in clinical settings [13]. Therefore, the feasibility rate of ultrasound-guided puncture may be higher in clinical practice when nurses use the film than under the experimental conditions of this study. In addition, the study did not include a comparison between the film used and commonly available plastic films, such as polyethylene or polyvinyl chloride (PVC) wraps, which have been widely used to prevent patient contamination during the COVID-19 pandemic. Third, both image quality and puncture feasibility were assessed subjectively, even though by experts. Future studies should consider more objective evaluation methods to minimize potential bias. Further research involving older adults and patients with medical conditions is warranted, as achieving reliable vascular access may be more clinically relevant than image quality alone in real-world settings.

## Conclusion

The quality of ultrasound images captured through a film specially designed for ultrasound guidance during blood vessel puncture was evaluated by an ultrasonographer at the right radial artery, forearm cephalic vein, and median cubital vein. This evaluation was conducted in comparison to images obtained without the film. The image quality with film was significantly lower than that of the images captured without it. However, clinical nurse assessments demonstrated that puncturability in the radial artery and forearm cephalic vein was comparable between cases with and without the use of the film.
